# Reciprocal Filial Piety Facilitates Academic Success via Autonomy: Generalizing Findings in Chinese Society to a Global Context

**DOI:** 10.3389/fpsyg.2020.00069

**Published:** 2020-02-07

**Authors:** Jing Zhou, Qingke Guo, Ruru Xu

**Affiliations:** ^1^Guangxi University and College Key Laboratory of Cognitive Neuroscience and Applied Psychology, Guangxi Normal University, Guilin, China; ^2^School of Psychology, Shandong Normal University, Jinan, China

**Keywords:** filial piety, autonomy, academic achievement, cross-culture, cultural universals

## Abstract

In cross-cultural psychology it is important to examine the universal (etic) and specific (emic) aspects of culture constructs. Filial piety is a core value of Chinese society that has shown related to psycho-social and academic development. This study was designed to investigate whether these relations revealed in specific cultural settings can be generalized to a global context. Using Chinese junior high school students as participants, Study 1 was intended to analyze the relations between filial piety and academic achievement, and whether autonomy need satisfaction serves as a bridge between them at the students’ and classes’ level. Study 2 was designed to examine whether these psycho-social and academic effects of filial piety can be applicable to a global context via analyzing two country-level databases [i.e., World Values Survey (WVS) and Program for International Student Assessment (PISA)]. The results of Study 1 showed that reciprocal filial piety was positively associated with academic achievement via the satisfaction of the need for autonomy, the authoritarian filial belief was negatively associated with academic achievement. The results of Study 2 showed that in a global context reciprocal filial belief in a society was related to the endorsement of autonomy, which in turn positively related to students’ academic achievement in that society, while authoritarian filial belief did not show such effects. These findings suggest that some psychological constructs established in non-Western settings can also be applied to a global context.

## Introduction

An important topic of cross-cultural study is whether psychological constructs established in Western cultures are applicable to non-Western settings (King and McInerney, 2014). In this study we went in the reverse direction. Specifically, the present study was intended to investigate whether the association between filial piety and academic achievement among Chinese students could be generalized to a global context. Filial piety is considered as a key virtue in Chinese society and other Confucian-heritage cultures (Hui et al., 2011; Cheah et al., 2018). For Chinese students academic success at schools is an important way to fulfill their filial beliefs (e.g., bring honor to the family). Thus, we firstly used a Chinese sample to examine whether filial piety was associated with academic achievement via autonomy need satisfaction. Then we used country-level databases to investigate whether the relationships among these research variables could be generalized to a global context. This may provide new evidence showing the universal (etic) and specific (emic) aspects of cultural constructs, especially the ones established in non-Western settings (King and McInerney, 2014).

### Filial Piety in Chinese Society

A salient feature of East Asian societies is a strong endorsement of filial piety, a value system stipulating children’s obligations to their parents and family elders (Ho, 1996; Yeh, 2003; Guo et al., 2017). Adult children are required to prioritize the interests of the family over their own, and provide material and emotional support to their parents (Ho, 1996). According to Yeh and Bedford (2004), filial attitudes toward the parents include a sense of owing and submission (refers to as the authoritarian filial piety), and a sense of gratitude and love (refers to as the reciprocal filial piety). These two aspects of filial piety were characterized by distinct parent–child relationships (Fuligni and Zhang, 2004) that have different implications for children’s psycho-social and academic development (Fuligni and Zhang, 2004; Pomerantz et al., 2011).

Specifically, reciprocal filial piety is characterized by natural intimate feelings and close relationships between parents and children, involving the benefaction of parents and gratitude of children (Yeh and Bedford, 2004). Children with reciprocal filial piety tend to repay their parents out of respect and love after they perceive parents’ efforts, support, and sacrifice for them (Yeh and Bedford, 2004). Previous research has found that this aspect of filial piety was positively associated with harmonious interpersonal relationships, psycho-social and behavioral development (e.g., perspective taking, self-disclosure behavior), as well as positive-oriented personality traits (openness, agreeableness, and extroversion) (Yeh and Bedford, 2003).

While authoritarian filial piety emphasizes hierarchy and submission, entailing children’s suppression of their needs in order to compliance with the family (Yeh and Bedford, 2004). Children are required to uphold honor for their families, take care of their parents, maintain family order, and continue family line by bearing male offspring. Obedience and indebtedness to parents are strongly emphasized because of their seniority. These characteristics of this aspect of filial piety usually lead to negative psychological outcomes (Yeh and Bedford, 2003), such as a higher level of depression, anxiety, and aggression (Yeh, 2006).

Beyond above-mentioned aspects, the academic effects of filial piety have also been investigated by previous researchers (Chen and Ho, 2012; Chen, 2016). As mentioned by Fuligni (2001), taking responsibility to family was an important source of motivation to study, especially for students from Asian families. Children tend to put more efforts on academic tasks when they view academic success as an important way to repay their parents, otherwise they may feel guilty (Fuligni, 2001). However, highly emphasizing honor for their family via academic achievement may also lead to unfavorable outcomes. For example, Ho (1996) suggested that authoritarian filial piety might lead to lower levels of creativity and cognitive inflexibility, which hindered academic achievement in the long run, in contrast, reciprocal filial piety generally related to positive-oriented development, such as flexible and resilient mindset, positive peer relationships at schools. Moreover, compared with less warm and coercive parent–child interactions that can lead to children’s poor development in social and cognitive domains (Conger et al., 1995), intimate parent–child relationships which are associated with reciprocal filial piety have great effects on inspiring adolescents’ involvement in academic tasks and facilitating better academic performance (Steinberg and Silk, 2002).

### Filial Piety in a Global Context

Endorsement of filial attitudes toward the parents is not only an Asian heritage. It can be found everywhere in the world (Poskaitë, 2014). Actually, repaying parents because of their efforts and resources invested in their children is deeply embedded across various cultures (Jones et al., 2011). The Christian doctrine and the teachings of Islam both require the adherents to respect and love their parents (Dykstra and Fokkema, 2012). For example, in Christian tradition, a child’s first obligation is to honor his/her father and mother. The Holy Bible says, “Listen to your father who gave your life, and don’t despise your mother when she is old” (Proverbs 23:22). Devotion and loyalty to family are imperative for individuals in Latin American cultures, with the needs of the family usually prioritize over the needs of the individuals (Fuligni, 2001). Overall, though filial obligations are also endorsed by adult children in individualist cultures, such as take care of their older parents (Dykstra and Fokkema, 2012), filial norms to parents are more highly valued in collectivist cultures (Fuligni et al., 1999; Fuligni, 2001; Pomerantz et al., 2011).

The relations between filial responsibility and psycho-social outcomes have been investigated in many cultural settings. For example, using Latino and American samples, Fuligni (2001) found that a higher sense of filial responsibility was generally associated with more positive psychological outcomes, such as emotional and psychological well-being. Research using the American adolescents from the multicultural (including Asian, Latin American, and European) backgrounds also shows that adolescents endorsing filial beliefs are more likely to develop positive interpersonal (e.g., family and peer) relationships and achieve greater academic success (Fuligni et al., 1999; Fuligni, 2001). Pomerantz et al. (2011) also found that the sense of filial responsibility to parents was predictive of academic engagement and academic outcomes in adolescents from both Chinese and American cultural backgrounds.

### Filial Beliefs, Autonomy, and Academic Achievement

The cultivation of autonomy has been strongly highlighted across different cultural settings given its promising benefits for individual’s self-development (specifically for adolescents), such as psycho-social adjustment, well-being, and academic outcomes (Ryan and Deci, 2000a; Van Petegem et al., 2012; Tam, 2016). As many theorists propose, autonomy can be regarded as an umbrella term including a wide range of psychological constructs, such as independence, self-endorsement, and agency (Beyers et al., 2003; Ryan et al., 2006), wherein independence and self-endorsement are now widely investigated in the literature (Van Petegem et al., 2012).

According to Self-Determination Theory (SDT; Ryan and Deci, 2000a), autonomy refers to volition or self-endorsement, individual with higher level of autonomy tend to feel that they are the master of their own destiny and life, and are more likely to actively engage in activities as their genuine interest and internal values rather than external pressure (Ryan and Deci, 2000a). Another important meaning of autonomy is independence, indicating the extent to which individual behave by themselves, which is opposed to rely on others (Smetana et al., 2004). Existing findings have indicated that the satisfaction of autonomy need can inspire individual’s internal motivation for engaging into activities and advance their development (Ryan and Deci, 2000b).

The effects of autonomy on academic outcomes have been investigated by many researchers (Patrick et al., 1993; Peters et al., 2007; Diseth et al., 2012). Findings have indicated that the fulfillment of autonomy can positively predict academic achievement. The mechanism underlying this relation is that the satisfaction of autonomy benefits for inspiring students to engage into learning as their own pursuits and interest instead of external pressure. If the students have positive attitudes toward learning, they may take more efforts to overcome academic challenges thereby achieving desirable academic outcomes (Diseth et al., 2012). Similar findings also have shown that students who experience greater autonomy at schools are more likely to have positive emotions and place more efforts on academic tasks (Maralani et al., 2016; Gutiérrez and Tomás, 2019).

Previous research suggested that harmonious and supportive parent–child relationships could be one necessary precondition for cultivating autonomy (Hurst, 2010; Liu, 2013). Reciprocal filial piety, characterized by intimate feelings and close relationships between parents and children, is conductive to children’s autonomy. Literature shows that close-knit relationship with parents is associated with children’s a greater sense of belonging to their parents (Bao and Lam, 2008; Hui et al., 2011). Children live in this intimate parent–child relationship tend to perceive parental involvement (such as parental aspiration and expectations) as support and encouragement, and thus facilitate them transform parents’ expectancy as personal goals and self-determination, which benefit for inspiring them actively engage into learning as pursuit of their own (Bao and Lam, 2008; Hui et al., 2011).

However, if children are socialized to suppress their needs in family interactions, and inhibit themselves to meet parents’ requirement and social criterion, their need for autonomy is less likely to be fulfilled (Yeh, 2006). Children may feel out of control, incompetence, helplessness, and frustration in their daily life, resulting in weaker academic motivation and poorer academic performance (Ho, 1996). These findings suggest that reciprocal filial piety that is associated with the fulfillment of autonomy, but not authoritarian filial piety, may be conductive to better academic performance.

### The Present Study

The main aim of this study is to investigate whether filial piety is associated with academic performance via autonomy need satisfaction, both in Chinese background and in a global context. In Study 1, we used Chinese junior high school students as participants. We constructed multilevel hierarchical linear modeling to examine the relations between filial beliefs, autonomy need satisfaction, and academic achievement at the students’ and classes’ level. Study 2 investigated the relationships among the above-mentioned variables using two open databases – the World Values Survey (WVS) and Program for International Student Assessment (PISA), which can be freely used by everyone. WVS contains items measuring filial obligation and the endorsement of autonomy at national level.

Filial piety includes two dimensions according to the model proposed by Yeh and Bedford (2003). According to the previous findings, we hypothesize that reciprocal filial belief can positively predict academic achievement, and this is true both in Chinese background (*hypothesis 1*) and in a global context (*hypothesis 2*). Authoritarian filial belief is not associated with academic achievement, and this is true both in Chinese background (*hypothesis 3*) and in a global context (*hypothesis 4*). In Study 1 autonomy need satisfaction was measured by a scale constructed by Deci and Ryan (2000) and Gagné (2003). Based on the existing findings, we hypothesize that in Chinese background the association between reciprocal filial piety and academic achievement can be explained by autonomy need satisfaction (*hypothesis 5*). Similarly, in Study 2 we hypothesize that the association between reciprocal filial piety and academic achievement at national level can be explained by the satisfaction of the need for autonomy (*hypothesis 6*).

Exploring the role of filial piety in facilitating academic performance is of great importance because enhancing family beliefs (especially reciprocal filial belief) may be an effective way to facilitate students’ motivation to learn. And this is particularly valuable based on the increased reports suggest that a substantial gradual decrease of academic motivation has been observed during later childhood and early adolescent across different cultural backgrounds (Gottfried et al., 2009; Bugler et al., 2016).

## Study 1

### Participants

Participants of Study 1 were 750 junior high school students (381 girls, *M*_age_ = 13.08 years, *SD*_age_ = 1.20) randomly recruited from 15 classes of public middle schools in Eastern China. There were about 50 participants in each class. Among them 35.06% were from Grade 7, 34% were from Grade 8, and 30.93% were from Grade 9; 35% came from city/town, 34% came from countryside, the rest came from the suburbs; 61% of them were the only child in their native family. Written informed consent were obtained from all participants and their parents, participants were encouraged to complete these measures as their real beliefs.

### Measures

#### Filial Beliefs

In Study 1, filial beliefs, including reciprocal filial belief and authoritarian filial belief, were measured by the Filial Piety Scale (FPS; Yeh and Bedford, 2003; Chen, 2014). FPS consists of 16 items using a 6-point scale ranging from 1 (extremely unimportant) to 6 (extremely important). The reciprocal filial belief dimension (e.g., be frequently concerned about my parents’ general well-being) and the authoritarian filial belief dimension (e.g., taken my parents’ suggestions even when I do not agree with them) each includes eight items. The total scores of all items of each dimension were taken to represent the levels of filial beliefs. FPS has showed acceptable reliability and validity in previous research using junior high students as participants (Yeh and Bedford, 2004). In this study, Cronbach’s alpha for reciprocal and authoritarian filial beliefs were 0.81 and 0.74, respectively.

#### Autonomy Need

In Study 1, the Basic Psychological Needs Scale (BPNS; Gagné, 2003) was used to assess the satisfaction of the need for autonomy. The Chinese version of BPNS includes 21 items and has been proved to be a reliable and valid measure of Chinese middle school students’ psychological needs (Zhen et al., 2017). In this study, the autonomy dimension was used to assess the satisfaction of the need for autonomy. A sample item is “I feel I am free to decide for myself how to live my life” (autonomy). Each item is rated on a 7-point scale (1 = strongly disagree, 7 = strongly agree). The total scores of all items were taken to represent the levels of autonomy. In this study, Cronbach’s alpha was 0.70.

#### Academic Achievement

In Study 1, the participants’ final grades in Reading, and Mathematics were obtained from school records. These scores can be used as valid measures of academic achievement (Chen et al., 2010). These scores were standardized according to grades, and were summed to create a total score of academic achievement.

### Results

#### Correlation Analysis

Pearson’s correlations among research variables were presented in Table 1. Reciprocal filial belief was positively and significantly associated with autonomy, and academic achievement, authoritarian filial belief was negatively associated with academic achievement. In addition, autonomy was also significantly associated with academic achievement.

**TABLE 1:** Correlations between filial beliefs, autonomy, and academic achievement (Study 1).

	**1**	**2**	**3**	**4**
1. Reciprocal filial belief	–			
2. Authoritarian filial belief	0.26**	–		
3. Autonomy	0.07*	0.03	–	
4. Academic achievement	0.20**	−0.10**	0.10**	–

*p < 0.05, **p < 0.01.

#### Multilevel Hierarchical Linear Analysis

The multilevel hierarchical linear modelings were constructed using the HLM 6.08 software to examine the relations between reciprocal filial piety, autonomy, and academic achievement at students’ and classes’ levels. Firstly, to examine the relations between reciprocal filial piety, autonomy, and academic achievement at the students’ level, we constructed model 1 (academic achievement = β_0_ + β_1_ reciprocal filial piety + β_2_ autonomy + *e*_1_) and model 2 (autonomy = β_0_ + β_1_ reciprocal filial piety + *e*_2_). The models include dependent [academic achievement (model 1) and autonomy (model 2)] and independent variables [reciprocal filial piety and autonomy (model 1) and reciprocal filial piety (model 2)], intercept (β_0_), slope (β_1_ and β_2_), and error (*e*_1_ and *e*_2_).

The results showed that reciprocal filial piety and autonomy significantly predicted academic achievement (model 1); moreover, reciprocal filial piety also significantly predicted autonomy (model 2), suggesting that reciprocal filial piety can positively predict academic achievement via autonomy (Table 2). In addition, following the same procedure, the results also indicated that the authoritarian filial piety negatively predicted academic achievement (β = −0.02, *t* = −2.76, *p* < 0.05), the relation between the authoritarian filial piety and autonomy was not significant (β = 0.02, *t* = 0.72, *p* > 0.05).

**TABLE 2:** The multilevel hierarchical linear analytical results without the second level variables.

**Models**	**Independent**	**Dependent**	**Coefficient and**		
	**variables**	**variables**	**significance**		
			**Coefficient**	**Error**	***t***
Model 1	RFP	Academic achievement	0.03	0.00	5.32***
	Autonomy		0.02	0.00	2.39***
Model 2	RFP	Autonomy	0.06	0.03	1.99*

*p < 0.05, ***p < 0.001.

Then, to examine the relations between reciprocal filial piety, autonomy, and academic achievement at the classes’ level, we constructed model 3 (β_0_ = γ_00_ + γ_0__1_ class + *r*_1_) and model 4 (β_1_ = γ_1__0_ + γ_11_ class + *r*_2_) based on model 1 and model 2, separately. The models examine the extent that class variable affects the above-mentioned variables. The results indicated that class variable did not interfere the relations between reciprocal filial piety (β = −0.00, *t* = −0.20, *p* > 0.05), autonomy (β = 0.00, *t* = 0.06, *p* > 0.05), and academic achievement. In addition, class variable also did not interfere the relation between reciprocal filial piety (β = 0.00, *t* = 0.35, *p* > 0.05) and autonomy. Finally, the results showed that the relations between the authoritarian filial piety, autonomy, and academic achievement were all not significant (*p*_S_ > 0.05).

At the first level, the 95% bias-corrected bootstrap confidence interval did not include zero when reciprocal filial piety was the predictor and academic achievement was the outcome variable [CI_directpath_: (0.019, 0.040); CI_indirectpath(viaautonomy)_: (0.001, 0.003)], in addition, the direct pathway from the authoritarian filial piety to academic achievement also did not include zero [CI: (−0.029, −0.005)].

**FIGURE 1:**
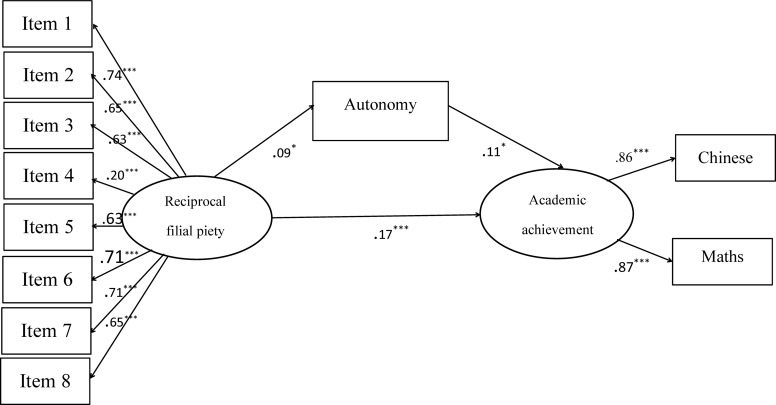
The1.1pc relationships between reciprocal filial belief, autonomy, and academic achievement (Study 1). ^∗^*p* < 0.05, ^∗∗∗^*p* < 0.001.

Additionally, the relationships between reciprocal filial belief, autonomy, and academic achievement were also examined through constructing the SEM (see Figure 1). The path model fit the data well (χ^2^/df = 1.73, NFI = 0.95, CFI = 0.98, TLI = 0.97, RMSEA = 0.03). Authoritarian filial belief was not significantly associated with autonomy need satisfaction and academic achievement, and therefore the results were not be represented in Figure 1 (Zhao et al., 2010).

## Study 2

### Participants

Participants of Study 2 were taken from WVS^1^ and PISA^2^. WVS, which has been conducted by the Executive Committee of the WVS Association since 1981, is a global research program focusing on human values and beliefs, such as politics importance, religion importance, and importance of equalizing chances for education. The WVS has been conducted six waves. Data were collected via random sampling method from nearly 100 countries/regions, representing 90% of the adult (18 years and older) population of the world. We used the participants who have finished the measures of this study. The date information of study 2 has been presented in the Appendix.

Program for International Student Assessment is a global academic assessment program aiming to test whether the adolescents have mastered the required knowledge and skills through a series of test, including Mathematics, Reading, and Science/Problem-solving examinations (Jerrim, 2015). PISA has been conducted by the Organization for Economic Co-operation and Development (OECD) in more than 60 countries/regions every 3 years since 2000. During the investigation, OECD randomly selected 4500–10,000 teenagers (15–16 years old) who were from different family backgrounds in each country. Additionally, a series of variables that reflect national-level economic, social, and population development were controlled in this study, including GDPpc^3^, HDI^4^, total fertility (live births per woman)^5^, and population density (see text footnote 3).

### Measures

#### Filial Beliefs

In Study 2, a FPS was constructed according to the definition of Yeh and Bedford (2003). The item in WVS “Respect and love for parents” (A025; 1 = always respect, 3 = neither) was used to measure reciprocal filial belief. This item parallels the one “grateful to my parents for raising me” in the reciprocity sub-scale of the FPS (Yeh, 2003; Chen, 2014). Dummy-variable method was used to translate this categorical variable into the continuous variable before into the regression analysis (Balestra, 2008). Another item “One of main goals in life has been to make my parents proud” (D054; 1 = agree strongly, 4 = strongly disagree) was used to measure authoritarian filial belief. This item parallels the one “meet my parents’ expectations” in the authoritarian sub-scale of the FPS (Yeh, 2003; Chen, 2014). These items were reverse-scored in order that higher scores indicate higher levels of reciprocal and authoritarian filial belief. Filial piety scores in each country/region were acquired by averaging the scores of all participants within that country/region.

#### Autonomy

In Study 2 the need for autonomy was indicated by the Autonomy Index in WVS (0 = obedience, 4 = determination/independence). The Autonomy Index comprises four binary choice items (0 = unmentioned, 1 = important), two positively worded (i.e., independence, determination) and two negatively worded (i.e., religious faith, obedience). It reflects how strongly independence and non-obedience are encouraged in a society. All participants were aggregated according to country/region, and the mean score of this item was used as an indicator of the satisfaction of the need for autonomy of each country/region. At national level Cronbach’s alpha of this measure is 0.75.

#### Academic Achievement

In Study 2, to chronologically match the six wave of WVS that was conducted since 1981, PISA scores of the last three waves (2009, 2012, and 2015) were used in this study. Reading and Mathematics scores were separately averaged and standardized according to countries/regions and waves, and eventually summed up as a final score of academic achievement at national level. GDP_pc_ (we used the data collected during 2010–2014), HDI (we used the data collected during 2013–2015), total fertility (live births per woman; we used the data collected during 2010–2015), and population density (we used the data collected in 2012) were used as controls in this study.

### Results

#### Correlation Analysis

Pearson’s correlations among research variables were presented in Table 3. The results showed that reciprocal filial belief was positively and significantly correlated with autonomy and academic achievement, and autonomy was significantly correlated with academic achievement, while authoritarian filial belief was not significantly associated with autonomy and academic achievement.

**TABLE 3:** Correlations between filial beliefs, autonomy, and academic achievement (Study 2).

	**1**	**2**	**3**	**4**
1. Reciprocal filial belief	–			
2. Authoritarian filial belief	0.14	–		
3. Autonomy	0.25*	0.11	–	
4. Academic achievement	0.37**	−0.07	0.34**	–

*p < 0.05, **p < 0.01.

#### Mediation Analysis

**FIGURE 2:**
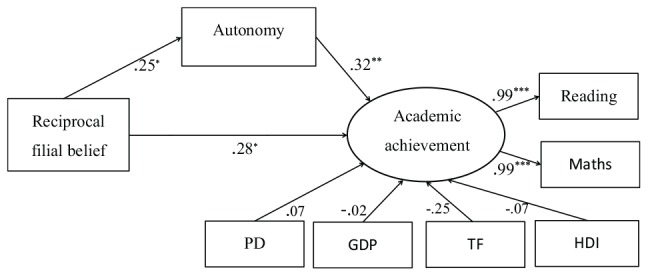
The relationships between reciprocal filial belief, autonomy, and academic achievement (Study 2). ^∗^*p* < 0.05, ^∗∗^*p* < 0.01, ^∗∗∗^*p* < 0.001. PD, population density; TF, total fertility (live births per woman); HDI, human development index.

Mediation analysis was carried out to examine the role of autonomy in the relationship between reciprocal filial belief and academic achievement. Reciprocal filial belief and autonomy were defined as observed variables, and academic achievement (measured by Reading and Mathematics) was defined as a latent variable (Figure 2).

The model fit the data well (χ^2^/df = 1.08, NFI = 0.95, CFI = 1.00, TLI = 0.99, RMSEA = 0.04). In Figure 2, reciprocal filial belief was significantly associated with autonomy, and autonomy was significantly associated with academic achievement after control variables were added to the model. The direct relation between reciprocal filial belief and academic achievement was also significant. The paths from control variables to academic achievement were all insignificant (*p*s > 0.05). The estimated mediating effect was 0.08, explaining 22.22% of the total effect (0.36) of reciprocal filial belief on academic achievement. The effects of authoritarian filial piety on autonomy and academic achievement were not significant (*p*s > 0.05).

We constructed bootstrap confidence intervals to test specific mediation and direct pathways in structural equation models. The 95% bias-corrected bootstrap confidence interval both did not contain zero when reciprocal filial piety was the predictor (CI_directpath_: 2.11–17.43; CI_indirectpath(viaautonomy)_: 0.01–6.53), while the 95% bias-corrected bootstrap confidence interval both contained zero when authoritarian filial piety was the predictor (CI_directpath_: −3.31–1.34; CI_indirectpath(viaautonomy)_: −0.28–3.24).

## Discussion

### Filial Beliefs, Autonomy, and Academic Achievement

In consistence with hypothesis 1, this study found that reciprocal filial belief can positively predicted Chinese adolescents’ academic achievement. This relationship still holds at national level, showing that a respectful and loving attitude toward the parents has positive implications for facilitating adolescents’ academic achievement at national level. Therefore, *hypothesis 2* was also supported. Difference from hypothesis 3, this study found that Chinese adolescents’ authoritarian filial belief was negatively and significantly associated with their academic achievement. In a global context, the results showed that a stronger endorsement of authoritarian filial piety in a country was not associated with adolescents’ higher levels of academic achievement in that country. Therefore, *hypothesis 4* was supported.

The results from this study indicated a positive and significant association between reciprocal filial piety and academic achievement both in Chinese society and in a global context. These findings highlight the importance of positive parent–child relationship that related to reciprocal filial piety in facilitating children’s psycho-social and academic development, suggesting that students who develop family obligations based on the reciprocity are more likely to obtain desirable academic outcomes (Leung and Zhang, 2000).

Our results also indicated that a general negative association between authoritarian filial piety and academic achievement both in Chinese society and in a global context. These findings suggest that if children are required to suppress their needs and feelings to meet their parents’ requirement and social criterion, their psycho-social and academic development are more likely to be hindered. This happen could be due to the fact that when children are demanded to repay their parents out of obedience and indebtedness (opposed to love and intimate affections), they tend to experience more negative emotions, such as a sense of losing control, incompetence, helplessness, and frustration, which hinder them from making positive-oriented changes (Yeh and Bedford, 2004).

In consistence with *hypothesis 5* and *hypothesis 6*, the results in this study also indicated that reciprocal filial piety was positively associated with the satisfaction of the need for autonomy, which in turn contributed to academic achievement both at individual level and at national level. This could be due to the harmonious parent–child relationship that related to reciprocal filial piety facilitates the fulfillment of autonomy and provides necessary nourishment for inspiring children to learn. For example, when parents encourage children’s active participation, acknowledge children’s perspectives, and provide social rewards for positive behaviors, children can naturally generate gratitude for their parents’ efforts and support, the expectations from parents in this close-knit family interaction process are more likely to be internalized as self-volition by children, inspiring them greater engagement into learning activities without experiencing external pressure or demands (Bao and Lam, 2008; Vasquez et al., 2016).

These results of this study have revealed culturally universal (i.e., etic) findings. Specifically, these findings are that: (1) Endorsing reciprocal filial piety in a society is beneficial for students’ academic development in that society, while endorsing authoritarian filial piety in a society does not have such effects. (2) The association between reciprocal filial piety and students’ academic development can be partly accounted by autonomy need satisfaction, and this is a universal finding across different cultural backgrounds.

### Strengths, Limitations, and Implications

This study throws new light on exploring the universal and specific aspects of psychological constructs. These findings in this study imply that filial beliefs established in non-Western settings can also be applied to a global context. The universality of filial piety may be the result of kin selection, an evolutionary strategy favoring reproductive success of relatives (Guo et al., 2017).

Nevertheless, some limitations of this study should be addressed. First, the participants and measures used in Study 1 and Study 2 were not equivalent. The participants in Study 1 were junior high school students, whereas the participants in Study 2 were samples from the population of 15 years and older. However, cultural values (e.g., filial beliefs, endorsement of personal autonomy) are broadly shared by various members in a society. So we assumed that the students’ attitudes toward filial piety can be reflected in other populations. Second, in Study 1 filial piety was measured by a scale with satisfying reliability and validity, while in Study 2 reciprocal and authoritarian filial piety were separately measured by only one item. Although one-item scale has been widely used in social surveys (Nevitte and Cochrane, 2006), whether these items can measure the target constructs is still a question. Furthermore, the autonomy index used in Study 2 indicated how strongly self-determination or independence (vs. obedience) was endorsed in a society, and we proposed that in a society where autonomy was strongly endorsed, the need for autonomy was more likely to be satisfied, whereas more direct evidence was needed to support this proposition. Third, the correlational study design of this study limits its power to infer causalities between research variables. Future researchers are encouraged to manipulate filial beliefs to examine their effects on academic performance. Fourth, in Chinese culture, individuating and relating autonomy may play different roles in motivating learning (Yeh and Yang, 2006; Yeh et al., 2009; Chen et al., 2013). The contribution of different forms of autonomy to academic success can be a meaningful theme that deserves to be investigated in the future research. Fifth, filial ideas endorsed by individual can also interact with other external factors and exert influence on academic achievement, future investigations can clarify the relations among these variables via advanced statistical methods (e.g., multilevel modeling). Last but not least, the results in this study showed that autonomy psychological need can partly mediate the association between filial piety and academic achievement, implying the existence of other mediators in this pathway, which are encouraged to be examined by future researchers.

This study highlights the role of family obligations in facilitating students’ academic achievement. This is particularly important because recently there is a decline in academic motivation during later childhood and early adolescent (Bugler et al., 2016). Family is the first agenda of socialization, to cultivate children’s family obligations, an authoritative parenting style rather than an authoritarian parenting style should be adopted by the parents. Parents should be more kind, loving, and caring to their children, the warm parenting style facilitates children repay to their parents with the same positive emotions, expectations from parents can be more easily to be internalized as self-pursuit rather than extra requirements by their children in the harmonious family interaction, which benefit for children’s academic engagement and performance.

## Data Availability Statement

Publicly available datasets were analyzed in this study. This data can be found here: https://www.cafonline.org.

## Ethics Statement

This study was carried out on the basis of the Declaration of Helsinki, and was conducted under the approval of the Institutional Review Board (IRB) at Shandong Normal University. Written informed consent was obtained from all participants before conducting the research, this study had also got the approval of volunteer participants’ guardians. This study caused no harm to participants’ physical and mental health.

## Author Contributions

JZ and QG designed the research, wrote, and revised the manuscript. RX and JZ collected and analyzed the data. All authors listed have made a substantial, direct and intellectual contribution to the work, and approved it for publication.

## Conflict of Interest

The authors declare that the research was conducted in the absence of any commercial or financial relationships that could be construed as a potential conflict of interest.

## Footnotes

1 http://www.worldvaluessurvey.org

2 http://www.oecd.org/pisa/keyfindings/

3 http://data.worldbank.org

4 http://hdr.undp.org

5 https://population.un.org

## References

[B1] Balestra P. (2008). Dummy variables. *New Palgrave Dictionary Econ.* 8 1452–1455.

[B2] Bao X. H. Lam S. F. (2008). Who makes the choice? Rethinking the role of autonomy and relatedness in Chinese children‘s motivation. *Child Dev.* 79 269–283. 10.1111/j.1467-8624.2007.01125.x 18366423

[B3] Beyers W. Goossens L. Vansant I. Moors E. (2003). A structural model of autonomy in middle and late adolescence: connectedness, separation, detachment, and agency. *J. Youth Adolescence* 32 351–365. 10.1023/a:1024922031510

[B4] Bugler M. McGeown S. St Clair-Thompson H. (2016). An investigation of gender and age differences in academic motivation and classroom behaviour in adolescents. *Educ. Psychol.* 36 1196–1218. 10.1080/01443410.2015.1035697

[B5] Cheah C. S. Leung C. Y. Ozdemir S. B. (2018). Chinese Malaysian Adolescents’ Social-cognitive reasoning regarding filial piety dilemmas. *Child Dev.* 89 383–396. 10.1080/01443410.2015.1008404 28105633

[B6] Chen B. Vansteenkiste M. Beyers W. Soenens B. Van Petegem S. (2013). Autonomy in family decision making for Chinese adolescents: disentangling the dual meaning of autonomy. *J. Cross Cult. Psychol.* 44 1184–1209. 10.1177/0022022113480038

[B7] Chen W. W. (2014). The relationship between perceived parenting style, filial piety, and life satisfaction in Hong Kong. *J. Fam. Psychol.* 28 308–314. 10.1037/a0036819 24821523

[B8] Chen W. W. (2016). The relations between filial piety, goal orientations and academic achievement in Hong Kong. *Educ. Psychol.* 36 898–915. 10.1080/01443410.2015.1008404

[B9] Chen W. W. Ho H. Z. (2012). The relation between perceived parental involvement and academic achievement: the roles of Taiwanese students’ academic beliefs and filial piety. *Int. J. Psychol.* 47 315–324. 10.1080/00207594.2011.630004 22288600

[B10] Chen X. Huang X. Chang L. Wang L. Li D. (2010). Aggression, social competence, and academic achievement in Chinese children: a 5-year longitudinal study. *Dev. Psychopathol.* 22 583–592. 10.1017/S0954579410000295 20576180

[B11] Conger R. D. Patterson G. R. Ge X. (1995). It takes two to replicate: a mediational model for the impact of parents’ stress on adolescent adjustment. *Child Dev.* 66 80–97. 10.2307/1131192 7497831

[B12] Deci E. L. Ryan R. M. (2000). The “what” and the “why” of goal pursuits: human needs and the self-determination of behavior. *Psychol. Inq.* 11 227–268. 10.1207/S15327965PLI1104_01 27055568

[B13] Diseth A. Danielsen A. G. Samdal O. (2012). A path analysis of basic need support, self-efficacy, achievement goals, life satisfaction and academic achievement level among secondary school students. *Educ. Psychol.* 32 335–354. 10.1080/01443410.2012.657159

[B14] Dykstra P. A. Fokkema T. (2012). Norms of filial obligation in the Netherlands. *Population* 67 97–122. 10.3917/popu.1201.0103

[B15] Fuligni A. J. (2001). Family obligation and the academic motivation of adolescents from Asian, Latin American, and European backgrounds. *New Direct. Child Adolescent Dev.* 2001 61–76. 10.1002/cd.31 11873482

[B16] Fuligni A. J. Tseng V. Lam M. (1999). Attitudes toward family obligations among American adolescents with Asian, Latin American, and European backgrounds. *Child Dev.* 70 1030–1044. 10.1111/1467-8624.00075

[B17] Fuligni A. J. Zhang W. (2004). Attitudes toward family obligation among adolescents in contemporary urban and rural China. *Child Dev.* 75 180–192. 10.1111/j.1467-8624.2004.00662.x 15015683

[B18] Gagné M. (2003). The role of autonomy support and autonomy orientation in prosocial behavior engagement. *Motiv. Emot.* 27 199–223. 10.1023/A:1025007614869

[B19] Gottfried A. E. Marcoulides G. A. Gottfried A. W. Oliver P. H. (2009). A latent curve model of parental motivational practices and developmental decline in math and science academic intrinsic motivation. *J. Educ. Psychol.* 101 729–739. 10.1037/a0015084

[B20] Guo Q. Li Y. Yu S. (2017). In-law and mate preferences in Chinese society and the role of traditional cultural values. *Evol. Psychol.* 15:1474704917730518. 10.1177/1474704917730518 28901196 PMC10481029

[B21] Gutiérrez M. Tomás J. M. (2019). The role of perceived autonomy support in predicting university students’ academic success mediated by academic self-efficacy and school engagement. *Educ. Psychol.* 39 729–748. 10.1080/01443410.2019.1566519

[B22] Ho D. Y. F. (1996). “Filial piety and its psychological consequences,” in *The Handbook of Chinese Psychology*, ed. Bond M. H. (Hong Kong: Oxford University Press), 155–165.

[B23] Hui E. K. Sun R. C. Chow S. S. Y. Chu M. H. T. (2011). Explaining Chinese students’ academic motivation: filial piety and self-determination. *Educ. Psychol.* 31 377–392. 10.1080/01443410.2011.559309

[B24] Hurst J. R. (2010). *The Development of Adolescent Autonomy: Contributions of the Mother-Child Attachment Relationship and Maternal Sensitivity.* Richardson, TX: The University of Texas at Dallas.

[B25] Jerrim J. (2015). Why do East Asian children perform so well in PISA? An investigation of Western-born children of East Asian descent. *Oxford Rev. Educ.* 41 310–333. 10.1080/03054985.2015.1028525

[B26] Jones P. S. Lee J. W. Zhang X. E. (2011). Clarifying and measuring filial concepts across five cultural groups. *Res. Nurs. Health* 34 310–326. 10.1002/nur.20444 21618557 PMC3155420

[B27] King R. B. McInerney D. M. (2014). Culture’s consequences on student motivation: capturing cross-cultural universality and variability through personal investment theory. *Educ. Psychol.* 49 175–198. 10.1080/00461520.2014.926813

[B28] Leung J. P. Zhang L. W. (2000). Modelling life satisfaction of Chinese adolescents in Hong Kong. *Int. J. Behav. Dev.* 24 99–104. 10.1080/016502500383520

[B29] Liu Y. L. (2013). Autonomy, filial piety, and parental authority: a two-year longitudinal investigation. *J. Genet. Psychol.* 174 557–581. 10.1080/00221325.2012.706660 24303573

[B30] Maralani F. M. Lavasani M. G. Hejazi E. (2016). Structural modeling on the relationship between basic psychological needs, academic engagement, and test anxiety. *J. Educ. Learn.* 5 44–54. 10.5539/jel.v5n4p44

[B31] Nevitte N. Cochrane C. (2006). Individualization in europe and america: connecting religious and moral values. *Comp. Sociol.* 5 203–230. 10.1163/156913306778667339

[B32] Patrick B. C. Skinner E. A. Connell J. P. (1993). What motivates children’s behavior and emotion? joint effects of perceived control and autonomy in the academic domain. *J. Pers. Soc. Psychol.* 65 781–791. 10.1037/0022-3514.65.4.781 8229650

[B33] Peters D. Jones G. Peters J. (2007). Approaches to studying, academic achievement and autonomy, in higher education sports students. *J. Hosp. Leisure Sport Tour.* 6 16–28. 10.3794/johlste.62.132

[B34] Pomerantz E. M. Qin L. Wang Q. Chen H. (2011). Changes in early adolescents’ sense of responsibility to their parents in the united states and china: implications for academic functioning. *Child Dev.* 82 1136–1151. 10.1111/j.1467-8624.2011.01588.x 21466541 PMC3134597

[B35] PoskaitëL. (2014). Filial Piety (xiao 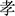 ) for the Contemporary and Global World. *Asian Stud.* 2 99–114. 10.4312/as.2014.2.1.99-114

[B36] Ryan R. M. Deci E. L. (2000a). Self-determination theory and the facilitation of intrinsic motivation, social development, and well-being. *Am. Psychol.* 55 68–78. 10.1037/0003-066x.55.1.68 11392867

[B37] Ryan R. M. Deci E. L. (2000b). The darker and brighter sides of human existence: basic psychological needs as a unifying concept. *Psychol. Inq.* 11 319–338. 10.1207/S15327965PLI1104_03

[B38] Ryan R. M. Deci E. L. Grolnick W. S. LaGuardia J. G. (2006). “The significance of autonomy and autonomy support in psychological development and psychopathology,” in *Developmental Psychopathology: Vol. 1: Theory and Methods*, 2nd Edn, eds Cicchetti D. Cohen D. (New York, NY: Wiley), 795–849. 10.1002/9780470939383.ch20

[B39] Smetana J. G. Campione-Barr N. Daddis C. (2004). Longitudinal development of family decision making: defining healthy behavioral autonomy for middle-class African American adolescents. *Child Dev.* 75 1418–1434. 10.1111/j.1467-8624.2004.00749.x 15369523

[B40] Steinberg L. Silk J. S. (2002). “Parenting adolescents,” in *Handbook of Parenting Vol. 1 Children and Parenting*, 2nd Edn, ed. Bornstein M. H. (New York, NY: Taylor & Francis), 103–133.

[B41] Tam J. (2016). Filial piety and academic motivation: high-achieving students in an international school in South Korea. *Int. J. Multicult. Educ.* 18 58–74. 10.18251/ijme.v18i3.1212

[B42] Van Petegem S. Beyers W. Vansteenkiste M. Soenens B. (2012). On the association between adolescent autonomy and psychosocial functioning: examining decisional independence from a self-determination theory perspective. *Dev. Psychol.* 48 76–88. 10.1037/a0025307 21910525

[B43] Vasquez A. C. Patall E. A. Fong C. J. Corrigan A. S. Pine L. (2016). Parent autonomy support, academic achievement, and psychosocial functioning: a meta-analysis of research. *Educ. Psychol. Rev.* 28 605–644. 10.1007/s10648-015-9329-z

[B44] Yeh K. H. (2003). “The beneficial and harmful effects of filial piety: an integrative analysis,” in *Progress in Asian Social Psychology: Conceptual and Empirical Contributions*, eds Yang K.-S. Hwang K.-K. Pederson P. Daibo I. (Santa Barbara, CA: Praeger), 67–82.

[B45] Yeh K. H. (2006). The impact of filial piety on the problem behaviors of culturally Chinese adolescents. *J. Psychol. Chin. Soc.* 7 237–257.

[B46] Yeh K. H. Bedford O. (2003). Filial piety: a test of the dual filial piety model. *Asian J. Soc. Psychol.* 6 215–228. 10.1046/j.1467-839X.2003.00122.x

[B47] Yeh K. H. Bedford O. (2004). Filial belief and parent-child conflict. *Int. J. Psychol.* 39 132–144. 10.1080/00207590344000312 22587548

[B48] Yeh K. H. Bedford O. Yang Y. J. (2009). A cross-cultural comparison of the coexistence and domain superiority of individuating and relating autonomy. *Int. J. Psychol.* 44 213–221. 10.1080/00207590701749146 22029497

[B49] Yeh K. H. Yang Y. J. (2006). Construct validation of individuating and relating autonomy orientations in culturally Chinese adolescents. *Asian J. Soc. Psychol.* 9 148–160. 10.1111/j.1467-839X.2006.00192.x

[B50] Zhao X. Lynch J. G. Chen Q. (2010). Reconsidering Baron and Kenny: myths and truths about mediation analysis. *Soc. Sci. Electr. Publ.* 37 197–206. 10.1086/651257

[B51] Zhen R. Liu R. D. Ding Y. Wang J. Liu Y. Xu L. (2017). The mediating roles of academic self-efficacy and academic emotions in the relation between basic psychological needs satisfaction and learning engagement among Chinese adolescent students. *Learn. Individ. Diff.* 54 210–216. 10.1016/j.lindif.2017.01.017

