# Supplementary material for: Reciprocal Filial Piety Facilitates Academic Success via Autonomy: Generalizing Findings in Chinese Society to a Global Context
Source: Front Psychol. 2020 Feb 7;11:69. doi: 10.3389/fpsyg.2020.00069 (PMC7025463; doi:10.3389/fpsyg.2020.00069)
Supplement: Supplementary file 1 [file Data_Sheet_1.doc]

Appendix . The data information of WVS, PISA, and controlled variables of study 2

| countries/regions | A029 | A039 | A040 | A042 | Auto-  nomy | AFP | RFP | R15 | M15 | M12 | R12 | M09 | R09 | Z_R_ | Z_M_ | PD | TF | HDI | GDPpc |
| --- | --- | --- | --- | --- | --- | --- | --- | --- | --- | --- | --- | --- | --- | --- | --- | --- | --- | --- | --- |
| Albania | 0.45 | 0.47 | 0.67 | 0.44 | 1.98 | 0.66 | 2.85 | 405.00 | 413.00 | 423.00 | 441.00 | 414.00 | 416.00 | -1.01 | -.97 | 104.00 | 1.71 | 0.76 | 42,147.94 |
| Algeria | 0.28 | 0.26 | 0.29 | 0.48 | 2.69 | 1.00 | 2.93 | 503.00 | 494.00 | 518.00 | 518.00 | 534.00 | 501.00 | .90 | .95 | 15.00 | 2.96 | 0.72 | 3,325.80 |
| Andorra | 0.48 | 0.35 | 0.93 | 0.62 | 1.61 | 0.66 | 2.85 | 485.00 | 497.00 | 494.00 | 499.00 | 490.00 | 494.00 | .58 | .53 | 181.00 | 2.20 | 0.88 | 2,599.00 |
| Argentina | 0.42 | 0.32 | 0.62 | 0.62 | 2.02 | 0.66 | 2.79 | 499.00 | 507.00 | 501.00 | 523.00 | 513.00 | 497.00 | .88 | .78 | 15.00 | 2.33 | 0.82 | 1,582.14 |
| Armenia | 0.31 | 0.49 | 0.79 | 0.80 | 1.63 | 0.66 | 2.93 | 527.00 | 516.00 | 536.00 | 538.00 | 519.00 | 521.00 | -1.73 | -1.82 | 100.00 | 1.72 | 0.75 | 1,472.02 |
| Australia | 0.53 | 0.39 | 0.80 | 0.66 | 1.62 | 0.66 | 2.74 | 407.00 | 377.00 | 376.00 | 388.00 | 365.00 | 370.00 | -.65 | -.46 | 3.00 | 1.89 | 0.94 | 3,270.02 |
| Azerbaijan | 0.59 | 0.45 | 0.81 | 0.81 | 1.33 | 0.66 | 2.92 | 494.00 | 531.00 | 523.00 | 511.00 | 526.00 | 508.00 | .22 | .15 | 97.00 | 2.09 | 0.74 | 16,290.46 |
| Bahrain | 0.40 | 0.27 | 0.65 | 0.81 | 1.88 | 0.66 | 2.85 | 432.00 | 441.00 | 445.00 | 438.00 | 442.00 | 442.00 | 1.37 | 1.11 | 1,827.00 | 2.12 | 0.84 | 36,586.81 |
| Bangladesh | 0.67 | 0.45 | 0.26 | 0.81 | 1.81 | 0.66 | 2.90 | 475.00 | 456.00 | 478.00 | 483.00 | 489.00 | 472.00 | -.12 | -.70 | 1,101.00 | 2.21 | 0.53 | 13,423.07 |
| Belarus | 0.33 | 0.39 | 0.87 | 0.68 | 1.73 | 0.66 | 2.83 | 427.00 | 400.00 | 409.00 | 411.00 | 387.00 | 405.00 | 1.13 | 1.66 | 46.00 | 1.65 | 0.79 | 6,921.72 |
| Bosnia | 0.56 | 0.46 | 0.76 | 0.57 | 1.66 | 0.66 | 2.91 | 459.00 | 423.00 | 448.00 | 475.00 | 421.00 | 449.00 | -1.32 | -1.58 | 90.00 | 1.31 | 0.77 | 3,517.74 |
| Brazil | 0.31 | 0.28 | 0.50 | 0.51 | 2.41 | 0.66 | 2.90 | 497.00 | 542.00 | 560.00 | 523.00 | 555.00 | 533.00 | -1.15 | -1.32 | 24.00 | 1.77 | 0.76 | 6,480.20 |
| Bulgaria | 0.42 | 0.50 | 0.83 | 0.78 | 1.47 | 0.66 | 2.89 | 425.00 | 390.00 | 394.00 | 394.00 | 371.00 | 402.00 | .28 | -.06 | 64.00 | 1.52 | 0.80 | 6,038.68 |
| Canada | 0.60 | 0.48 | 0.69 | 0.70 | 1.52 | 0.66 | 2.78 | 487.00 | 464.00 | 479.00 | 477.00 | 447.00 | 474.00 | -1.03 | -.67 | 3.00 | 1.60 | 0.93 | 50,972.00 |
| Chile | 0.41 | 0.41 | 0.57 | 0.50 | 2.12 | 0.66 | 2.87 | 443.00 | 437.00 | 432.00 | 393.00 | 427.00 | 424.00 | .45 | .42 | 22.00 | 1.85 | 0.84 | 14,516.98 |
| China | 0.68 | 0.29 | 0.98 | 0.86 | 1.18 | 0.66 | 2.83 | 487.00 | 492.00 | 490.00 | 488.00 | 483.00 | 486.00 | .57 | .64 | 139.00 | 1.64 | 0.73 | 6,187.90 |
| Colombia | 0.30 | 0.21 | 0.54 | 0.49 | 2.56 | 0.66 | 2.91 | 519.00 | 520.00 | 561.00 | 545.00 | 541.00 | 536.00 | 1.47 | 1.44 | 39.00 | 1.90 | 0.74 | 7,327.72 |
| Cyprus | 0.45 | 0.43 | 0.60 | 0.56 | 1.96 | 0.66 | 2.85 | 499.00 | 493.00 | 493.00 | 483.00 | 487.00 | 489.00 | .52 | .47 | 121.00 | 1.38 | 0.87 | 13,677.26 |
| Czech Rep. | 0.21 | 0.39 | 0.91 | 0.82 | 1.67 | 0.66 | 2.72 | 401.00 | 404.00 | 388.00 | 404.00 | 377.00 | 385.00 | -1.54 | -1.49 | 129.00 | 1.48 | 0.88 | 20,100.60 |
| Dominican Rep. | 0.40 | 0.27 | 0.41 | 0.49 | 2.43 | 0.66 | 2.86 | 509.00 | 506.00 | 514.00 | 508.00 | 507.00 | 500.00 | .86 | .82 | 97.00 | 2.45 | 0.80 | 5,770.58 |
| Ecuador | 0.37 | 0.25 | 0.51 | 0.40 | 2.46 | 1.00 | 2.85 | 467.00 | 454.00 | 449.00 | 446.00 | 468.00 | 459.00 | -.20 | -.19 | 53.00 | 2.56 | 0.75 | 5,557.20 |
| El Salvador | 0.36 | 0.14 | 0.33 | 0.38 | 2.80 | 1.00 | 2.94 | 527.00 | 548.00 | 531.00 | 509.00 | 514.00 | 515.00 | 1.11 | 1.25 | 289.00 | 2.17 | 0.69 | 3,740.26 |
| Estonia | 0.54 | 0.61 | 0.87 | 0.52 | 1.46 | 0.66 | 2.83 | 482.00 | 488.00 | 477.00 | 488.00 | 496.00 | 470.00 | .08 | .36 | 28.00 | 1.59 | 0.85 | 17,507.78 |
| Ethiopia | 0.79 | 0.30 | 0.59 | 0.61 | 1.71 | 0.66 | 2.85 | 470.00 | 477.00 | 482.00 | 463.00 | 497.00 | 477.00 | .30 | .40 | 81.00 | 4.85 | 0.40 | 6,000.26 |
| Finland | 0.63 | 0.54 | 0.88 | 0.70 | 1.25 | 0.33 | 2.59 | 397.00 | 386.00 | 376.00 | 403.00 | 360.00 | 371.00 | -1.68 | -1.80 | 16.00 | 1.77 | 0.91 | 48,651.98 |
| France | 0.37 | 0.55 | 0.91 | 0.59 | 1.58 | 0.33 | 2.85 | 521.00 | 504.00 | 504.00 | 512.00 | 494.00 | 497.00 | .96 | .66 | 119.00 | 1.98 | 0.91 | 42,147.94 |
| Georgia | 0.54 | 0.30 | 0.49 | 0.78 | 1.88 | 0.66 | 2.91 | 479.00 | 470.00 | 471.00 | 485.00 | 477.00 | 468.00 | .24 | .12 | 66.00 | 2.00 | 0.75 | 3,325.80 |
| Germany | 0.68 | 0.55 | 0.89 | 0.86 | 2.65 | 0.66 | 2.58 | 516.00 | 532.00 | 573.00 | 542.00 | 546.00 | 539.00 | .28 | .42 | 228.00 | 1.43 | 0.92 | 16,290.46 |
| Ghana | 0.33 | 0.45 | 0.28 | 0.22 | 2.72 | 1.00 | 2.85 | 408.00 | 380.00 | 386.00 | 399.00 | 368.00 | 372.00 | -1.62 | -1.72 | 104.00 | 4.18 | 0.53 | 1,582.14 |
| Guatemala | 0.27 | 0.22 | 0.42 | 0.53 | 2.57 | 1.00 | 2.85 | 517.00 | 524.00 | 521.00 | 516.00 | 515.00 | 506.00 | 1.03 | 1.04 | 127.00 | 3.19 | 0.64 | 3,270.02 |
| Hong Kong | 0.46 | 0.22 | 0.94 | 0.91 | 1.47 | 0.66 | 2.85 | 488.00 | 482.00 | 487.00 | 488.00 | 501.00 | 483.00 | .44 | .45 | 6,522.00 | 1.20 | 0.92 | 36,586.81 |
| Hungary | 0.59 | 0.37 | 0.87 | 0.69 | 1.47 | 0.66 | 2.73 | 347.00 | 396.00 | 368.00 | 384.00 | 331.00 | 314.00 | -2.61 | -1.97 | 107.00 | 1.33 | 0.84 | 13,423.07 |
| India | 0.49 | 0.40 | 0.59 | 0.41 | 2.11 | 0.66 | 2.86 | 472.00 | 478.00 | 481.00 | 498.00 | 460.00 | 476.00 | .34 | .12 | 362.00 | 2.40 | 0.58 | 1,472.02 |
| Indonesia | 0.80 | 0.42 | 0.09 | 0.47 | 2.22 | 0.66 | 2.90 | 481.00 | 486.00 | 484.00 | 488.00 | 483.00 | 481.00 | .37 | .34 | 128.00 | 2.45 | 0.68 | 3,517.74 |
| Iran | 0.58 | 0.29 | 0.29 | 0.63 | 2.20 | 0.66 | 2.89 | 509.00 | 544.00 | 538.00 | 509.00 | 527.00 | 524.00 | 1.05 | 1.35 | 47.00 | 1.91 | 0.74 | 6,480.20 |
| Iraq | 0.30 | 0.21 | 0.12 | 0.27 | 3.05 | 1.00 | 2.85 | 447.00 | 479.00 | 466.00 | 486.00 | 445.00 | 464.00 | -.02 | -.06 | 70.00 | 4.25 | 0.77 | 6,038.68 |
| Italy | 0.58 | 0.44 | 0.66 | 0.74 | 1.46 | 0.66 | 2.85 | 423.00 | 408.00 | 391.00 | 410.00 | 388.00 | 398.00 | .91 | 1.03 | 203.00 | 1.42 | 0.90 | 35,907.00 |
| Japan | 0.67 | 0.60 | 0.94 | 0.94 | 1.58 | 2.69 | 2.74 | 416.00 | 420.00 | 427.00 | 441.00 | 419.00 | 421.00 | -1.24 | -1.38 | 335.00 | 1.41 | 0.93 | 42,124.06 |
| Jordan | 0.36 | 0.25 | 0.17 | 0.44 | 1.46 | 1.00 | 2.94 | 503.00 | 512.00 | 511.00 | 508.00 | 487.00 | 500.00 | -.90 | -.86 | 71.00 | 3.40 | 0.74 | 4,911.26 |
| Kazakhstan | 0.44 | 0.45 | 0.90 | 0.67 | 1.54 | 1.00 | 2.85 | 427.00 | 418.00 | 388.00 | 396.00 | 405.00 | 390.00 | .82 | .71 | 6.00 | 2.67 | 0.77 | 16,290.46 |
| Kuwait | 0.42 | 0.28 | 0.26 | 0.44 | 1.33 | 1.00 | 2.85 | 513.00 | 502.00 | 499.00 | 493.00 | 503.00 | 495.00 | 1.00 | .73 | 146.00 | 2.10 | 0.83 | 45,647.46 |
| Kyrgyzstan | 0.59 | 0.52 | 0.76 | 0.62 | 2.60 | 1.00 | 2.94 | 398.00 | 387.00 | 375.00 | 396.00 | 431.00 | 362.00 | .75 | .67 | 28.00 | 3.30 | 0.71 | 12,299.22 |
| Latvia | 0.38 | 0.48 | 0.86 | 0.81 | 1.88 | 0.66 | 2.79 | 498.00 | 492.00 | 500.00 | 512.00 | 543.00 | 495.00 | -1.79 | -1.34 | 34.00 | 1.50 | 0.83 | 14,111.06 |
| Libya | 0.38 | 0.25 | 0.21 | 0.41 | 1.91 | 1.00 | 2.85 | 402.00 | 402.00 | 407.00 | 441.00 | 371.00 | 404.00 | .77 | .87 | 3.75 | 2.45 | 0.80 | 16,290.46 |
| Lithuania | 0.72 | 0.36 | 0.78 | 0.77 | 1.48 | 0.66 | 2.80 | 434.00 | 444.00 | 434.00 | 442.00 | 419.00 | 425.00 | -.73 | -.66 | 54.00 | 1.59 | 0.84 | 14,560.86 |
| Malaysia | 0.75 | 0.37 | 0.38 | 0.75 | 1.75 | 1.00 | 2.85 | 495.00 | 494.00 | 485.00 | 490.00 | 466.00 | 483.00 | -.08 | -.12 | 87.00 | 2.11 | 0.79 | 16,290.46 |
| Mexico | 0.38 | 0.31 | 0.59 | 0.48 | 2.45 | 0.66 | 2.83 | 453.00 | 475.00 | 453.00 | 477.00 | 453.00 | 459.00 | .95 | .91 | 58.00 | 2.29 | 0.81 | 9,748.78 |
| Moldova | 0.46 | 0.29 | 0.62 | 0.68 | 2.24 | 0.66 | 2.90 | 505.00 | 510.00 | 518.00 | 523.00 | 512.00 | 501.00 | .62 | .37 | 127.00 | 1.28 | 0.68 | 2,025.28 |
| Montenegro | 0.54 | 0.42 | 0.85 | 0.58 | 1.95 | 0.66 | 2.89 | 496.00 | 486.00 | 489.00 | 504.00 | 482.00 | 484.00 | .56 | .71 | 48.00 | 1.71 | 0.80 | 6,977.54 |
| Morocco | 0.44 | 0.21 | 0.22 | 0.54 | 1.61 | 1.00 | 2.98 | 500.00 | 494.00 | 491.00 | 489.00 | 525.00 | 487.00 | .60 | 1.06 | 72.00 | 2.60 | 0.62 | 2,960.82 |
| Netherlands | 0.63 | 0.37 | 0.88 | 0.69 | 2.60 | 0.66 | 2.85 | 492.00 | 521.00 | 506.00 | 490.00 | 536.00 | 499.00 | -1.18 | -1.11 | 406.00 | 1.73 | 0.93 | 51,077.84 |
| New Zealand | 0.54 | 0.45 | 0.85 | 0.77 | 1.43 | 0.66 | 2.64 | 409.00 | 415.00 | 410.00 | 422.00 | 403.00 | 408.00 | -1.18 | -1.15 | 16.00 | 2.04 | 0.92 | 38,318.45 |
| Nigeria | 0.26 | 0.31 | 0.29 | 0.31 | 1.53 | 1.00 | 2.90 | 427.00 | 417.00 | 413.00 | 424.00 | 386.00 | 412.00 | -.65 | -.68 | 168.00 | 5.74 | 0.49 | 16,290.46 |
| Norway | 0.89 | 0.39 | 0.90 | 0.73 | 1.40 | 0.33 | 2.52 | 428.00 | 420.00 | 421.00 | 398.00 | 381.00 | 413.00 | .79 | .66 | 14.00 | 1.82 | 0.95 | 97,996.10 |
| Pakistan | 0.23 | 0.32 | 0.19 | 0.56 | 1.40 | 1.00 | 2.94 | 434.00 | 427.00 | 440.00 | 449.00 | 428.00 | 429.00 | .71 | .37 | 233.00 | 3.78 | 0.54 | 1,231.31 |
| Palestine | 0.43 | 0.20 | 0.24 | 0.52 | 2.62 | 1.00 | 2.85 | 485.00 | 490.00 | 482.00 | 475.00 | 493.00 | 478.00 | -.74 | -.75 | 233.00 | 4.25 | 0.77 | 2,599.00 |
| Peru | 0.30 | 0.25 | 0.51 | 0.45 | 2.69 | 0.66 | 2.92 | 498.00 | 492.00 | 515.00 | 509.00 | 495.00 | 500.00 | .83 | 1.17 | 23.00 | 2.40 | 0.76 | 6,069.58 |
| Philippines | 0.61 | 0.32 | 0.39 | 0.57 | 2.48 | 1.00 | 2.95 | 497.00 | 470.00 | 495.00 | 505.00 | 492.00 | 494.00 | .50 | .29 | 339.00 | 3.05 | 0.69 | 2,556.44 |
| Poland | 0.39 | 0.22 | 0.57 | 0.56 | 2.11 | 0.66 | 2.94 | 437.00 | 418.00 | 439.00 | 436.00 | 427.00 | 426.00 | -1.01 | -1.19 | 123.00 | 1.33 | 0.84 | 13,518.96 |
| Puerto Rico | 0.37 | 0.23 | 0.29 | 0.46 | 2.20 | 1.00 | 2.94 | 487.00 | 495.00 | 535.00 | 516.00 | 529.00 | 520.00 | 1.45 | 1.62 | 438.00 | 1.50 | 0.77 | 17,125.55 |
| South Korea | 0.61 | 0.40 | 0.80 | 0.86 | 2.77 | 0.66 | 2.93 | 509.00 | 495.00 | 519.00 | 524.00 | 498.00 | 503.00 | -1.37 | -1.22 | 495.00 | 1.23 | 0.65 | 24,945.87 |
| Taiwan | 0.74 | 0.32 | 0.91 | 0.82 | 1.22 | 0.66 | 2.93 | 500.00 | 511.00 | 501.00 | 481.00 | 487.00 | 496.00 | -1.12 | -1.42 | 643.00 | 1.11 | 0.77 | 20,333.00 |

Note: RFP=reciprocal filial piety; AFP=authoritarian filial piety; R=reading; M=Math; PD=population density; TF=Total fertility (live births per woman; HDI=human development index
